# Validation of a non-invasive prenatal test for fetal RhD, C, c, E, K and Fy^a^ antigens

**DOI:** 10.1038/s41598-023-39283-3

**Published:** 2023-08-07

**Authors:** Brian Alford, Brian P. Landry, Sarah Hou, Xavier Bower, Anna M. Bueno, Drake Chen, Brooke Husic, David E. Cantonwine, Thomas F. McElrath, Jacqueline A. Carozza, Julia Wynn, Jennifer Hoskovec, Kathryn J. Gray

**Affiliations:** 1 BillionToOne, Inc., 1035 O’Brien Drive, Menlo Park, CA 94025 USA; 2 Division of Maternal-Fetal Medicine, Brigham and Women’s Hospital and Harvard Medical School, Boston, MA USA

**Keywords:** Genetics, Genetic testing

## Abstract

We developed and validated a next generation sequencing-(NGS) based NIPT assay using quantitative counting template (QCT) technology to detect RhD, C, c, E, K (Kell), and Fy^a^ (Duffy) fetal antigen genotypes from maternal blood samples in the ethnically diverse U.S. population. Quantitative counting template (QCT) technology is utilized to enable quantification and detection of paternally derived fetal antigen alleles in cell-free DNA with high sensitivity and specificity. In an analytical validation, fetal antigen status was determined for 1061 preclinical samples with a sensitivity of 100% (95% CI 99–100%) and specificity of 100% (95% CI 99–100%). Independent analysis of two duplicate plasma samples was conducted for 1683 clinical samples, demonstrating precision of 99.9%. Importantly, in clinical practice the no-results rate was 0% for 711 RhD-negative non-alloimmunized pregnant people and 0.1% for 769 alloimmunized pregnancies. In a clinical validation, NIPT results were 100% concordant with corresponding neonatal antigen genotype/serology for 23 RhD-negative pregnant individuals and 93 antigen evaluations in 30 alloimmunized pregnancies. Overall, this NGS-based fetal antigen NIPT assay had high performance that was comparable to invasive diagnostic assays in a validation study of a diverse U.S. population as early as 10 weeks of gestation, without the need for a sample from the biological partner. These results suggest that NGS-based fetal antigen NIPT may identify more fetuses at risk for hemolytic disease than current clinical practice, which relies on paternal genotyping and invasive diagnostics and therefore is limited by adherence rates and incorrect results due to non-paternity. Clinical adoption of NIPT for the detection of fetal antigens for both alloimmunized and RhD-negative non-alloimmunized pregnant individuals may streamline care and reduce unnecessary treatment, monitoring, and patient anxiety.

## Introduction

Hemolytic disease of the fetus and newborn (HDFN) is a serious blood disorder primarily caused by the destruction of a fetus’s or neonate’s red blood cells (RBCs) by immunoglobulin G (IgG) antibodies of the alloimmunized pregnant person. Alloimmunization refers to the production of antibodies to foreign cells that gain access to an individual’s circulation, most commonly occurring with pregnancy or blood transfusions. HDFN can cause severe fetal anemia which, in turn, may lead to fetal hydrops, heart failure, hyperbilirubinemia and associated kernicterus (toxic levels of bilirubin in the brain), and perinatal death.

Historically, the most common cause of HDFN was Rhesus factor (Rh) incompatibility (i.e., when an RhD-negative pregnant person is exposed to antigens from an RhD-positive fetus). However, the introduction of Rho(D) immune globulin in the late 1960s drastically reduced the rates of alloimmunization resulting from Rh incompatibility. RhD sensitization (the development of maternal IgG antibodies against RhD-positive RBCs) and RhD-associated HDFN has decreased from 16% to less than 1% of RhD-negative pregnant individuals in present day^1^. The current clinical standard of care in the U.S. is to administer prophylactic Rho(D) immune globulin to all RhD-negative pregnant people at 26–28 weeks gestation, prior to any invasive procedure or following any potential sensitization event (e.g., trauma), and at delivery if the neonate is RhD-positive to prevent sensitization. However, this intervention is unnecessary in approximately 40% of RhD-negative pregnant individuals who carry an RhD-negative fetus. Additionally, decreasing the need for Rho(D) immune globulin is desirable for the many parts of the world where there are Rho(D) immune globulin shortages^2^.

In addition to RhD, other RBC antigens are associated with HDFN, with approximately 0.5–1% of pregnancies overall complicated by alloimmunization^3^. The risk of alloimmunization is greater among populations that receive regular blood transfusions, such as individuals with sickle cell disease^4–6^. In the U.S., the standard of care is antibody screening in the first trimester of pregnancy and, if the screen detects HDFN-associated antibodies, additional testing is indicated to determine the HDFN risk for the fetus^3^. This includes serological or genetic evaluation of the partner, when possible, and amniocentesis to determine fetal antigen genotype. Determination of fetal antigen genotype reduces unnecessary monitoring and treatment of pregnancies with concordant antigen status that are not at risk for HDFN. However, barriers to partner antigen screening, and concerns about the risk of amniocentesis, leads to lack of knowledge about the fetal antigen status in many pregnancies which then must be treated as high-risk for HDFN. In approximately 35–50% of pregnancies with alloimmunization the fetus is antigen positive and at risk for HDFN; however, in the remaining pregnancies, the fetus is negative for the corresponding antigen and therefore is not at risk^6^.

Non-invasive prenatal testing (NIPT) of cfDNA can be used to determine fetal antigen genotype for the prediction of fetal antigen phenotype. Many European countries have adopted this practice to determine who receives Rho(D) immune globulin and to guide pregnancy management of alloimmunized pregnant individuals^4,7–11^. However, European-based assays are logistically challenging for U.S.-based patients, often require testing to be delayed until after 20 weeks gestation, and have high rates of inconclusive results, particularly for people of non-European ancestry^1,4,12–14^.

A challenge of the NIPT assays used in Europe is that they employ qualitative polymerase chain reaction (PCR) technology under the assumption that an RhD-negative fetus will be homozygous for the *RHD* gene deletion, which is the most common genotype that results in an RhD-negative phenotype^15^. However, up to 30% of RhD-negative Black individuals in the U.S., and 60% of RhD-negative Africans, have non-deletion *RHD* gene variants, such as *RHDΨ*, which would not be detected by such an assay^15,16^. Other assays may identify a non-deletion *RHD* gene variant but cannot determine the fetal RhD status to guide the administration of Rho(D) immune globulin^15,16^. Additionally, qualitative NIPT assays do not directly measure the fraction of cfDNA of fetal origin, but rather rely on the detection of Y chromosome genes or a single autosomal reference gene to confirm the presence or absence of fetal cfDNA. This strategy results in a fetal sex bias inaccuracy and an increased potential for false negatives, especially if tested too early in pregnancy when the fraction of cfDNA of fetal origin is lower^17–19^.

The American College of Obstetricians and Gynecologists (ACOG) noted that NIPT could be an effective and attractive strategy for the management of RhD-negative and alloimmunized pregnancies if the inclusivity, feasibility, and cost-effectiveness of cfDNA tests are addressed for the U.S. population^1^. The European SNP-based NIPT assays result in greater occurrence of no results or inconclusive results for people of non-European ancestry because of the inability to predict fetal RhD-status in the presence of a non-deletion *RHD* variant, resulting in a less efficient and more costly strategy than prophylactic Rho(D) immune globulin for all RhD-negative pregnancies if the qualitative assay was implemented in the U.S. population^12,20,21^. The application of the recently developed quantitative counting template (QCT) technology to NIPT has the potential to address these barriers of inclusivity and feasibility for the U.S. population and be more cost-effective by eliminating unnecessary prophylactic Rho(D) immune globulin. The technology is capable of both accurately quantifying genes of interest as well as sequence variants using next generation sequencing (NGS). QCT technology has previously been validated for NIPT of autosomal recessive conditions in cfDNA^22–24^.

Here we describe the application of QCT technology NIPT for the detection of the fetal genotypes that encode for RhD, C, c, E, K (Kell), and Fy^a^ (Duffy). These antigens are all associated with HDFN and can be detected as early as 10 weeks gestation. This performance is achieved by the combination of QCT technology with genome-wide assessment of polymorphic locations that determine the expected cfDNA fetal molecule count^22^. These quantitative measurements are critical to ensure there is sufficient fetal cfDNA present in the sample and significantly reduce the false negative rate. Moreover, the quantitative sequencing-based design of the fetal antigen NIPT described herein enables the detection of fetal antigen status for common genotypes as well as non-deletion *RHD* gene variants, thus improving clinical utility for the diverse U.S. population and avoiding the necessity of a partner.

## Methods

### Assay overview and design

Unless otherwise indicated, the fetal antigen assay is performed using a single tube of 5–10 mL of whole blood collected from the pregnant person. Plasma is isolated from the blood and cfDNA is extracted using a bead-based extraction kit.

**Figure 1:**
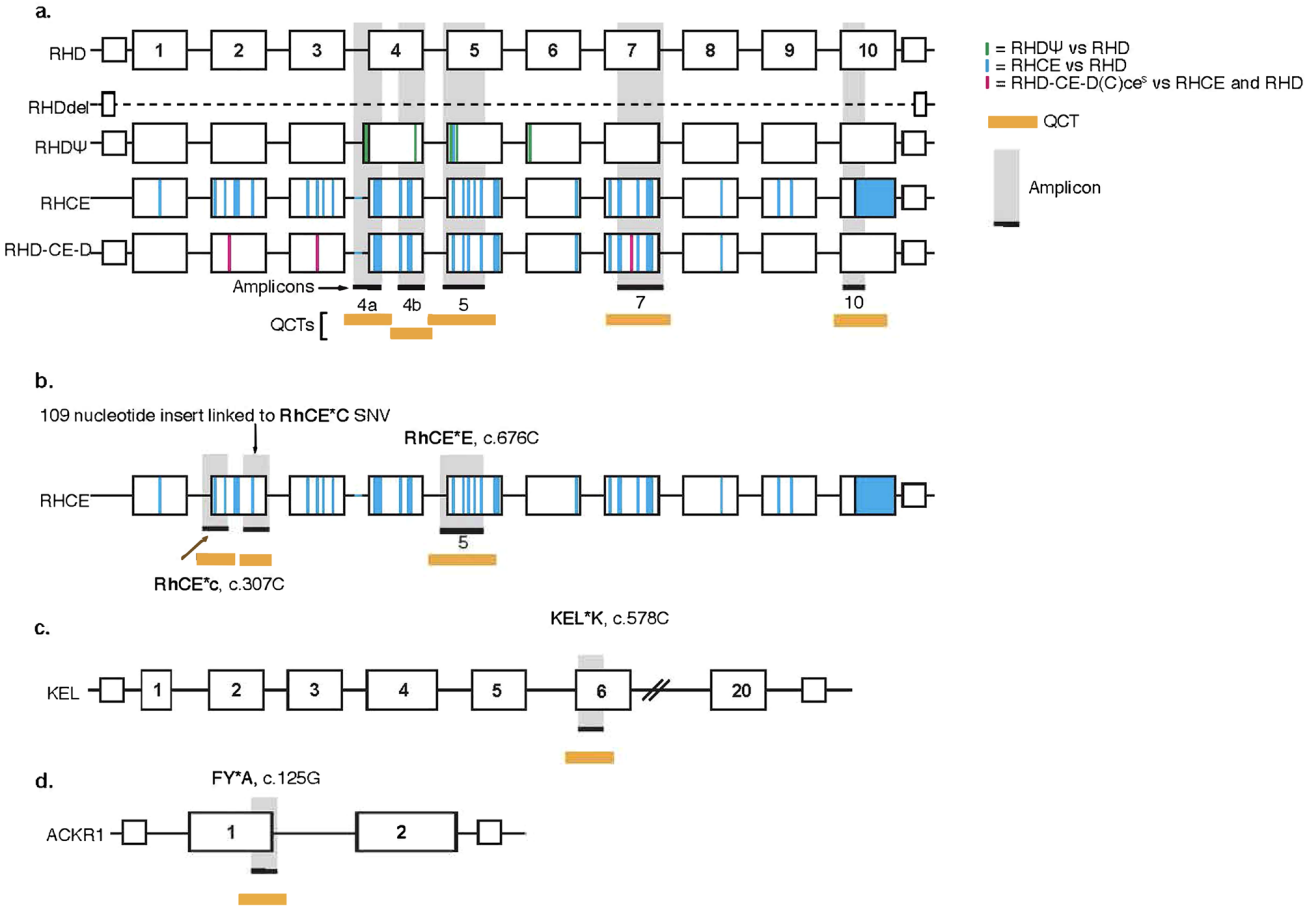
Schematic of the genes associated with the antigens, amplicons, and quantitative counting templates (QCTs). QCTs quantify the molecular count for each genomic loci. (**a**) The wild type *RHD* gene is shown along with its common variants, *RHD* gene deletion and *RhDΨ* variant, and the homologous *RHCE* gene and *RHD-CE-D* hybrid gene. Differences between these variants and homolog are highlighted as indicated in the legend. (**b**) Schematic of the *RHCE* gene, single nucleotide variants (SNVs) associated with RhCE*C (c.307C), RhCE*c (c.307T) and RhCE*E (c.676C) genotypes. The amplicon for the RHCE*C quantifies a 109 nucleotide insert highly linked to RhCE*C. The RhCE*E (c.676C) is located in the in amplicon 5 of the RhD assay (**c**) Schematic of the *KEL* gene and KEL*K (c.578C) SNV. (**d**) Schematic of the *ACKR1* gene and FY*A (c.125G) SNV. The primer details for the amplicons are in Table S1.

To detect the genotype of fetal antigens RhD, C, c, E, K, and Fy^a^ in pregnant individuals, a highly-multiplexed PCR-based NIPT assay with a NGS readout that employs QCT technology was developed^22^. The assay is comprised of five amplicons across the *RHD* gene that amplifies regions that are unique between the wild-type *RHD* gene, the *RHCE* homolog gene (exons 7 and 10) and the *RHDΨ* pseudogene (exons 4 and 5) (Fig. 1a, Table S1)^16,25^.

The remaining antigens (C, c, E, K, and Fy^a^) result from single nucleotide variants (SNVs) RhCE*C, RhCE*c, RhCE*E, KEL*K and FY*A, respectively. A single amplicon was designed for each SNV based on previously published antigen genotyping assays for antigen phenotype prediction (Table S1). Specifically,

- the amplicon for the C antigen phenotype quantifies a 109-nucleotide insert linked to SNV RhCE*C^11^ (Fig. 1b),
- the amplicon for the c antigen phenotype directly amplifies the SNV RhCE*c^9^ (Fig. 1b),
- the RhCE*E SNV^11^, which corresponds to the E antigen phenotype, is located in amplicon 5 of the RhD antigen assay (Fig. 1b), and
- the K and Fy^a^ amplicons target the SNVs that determine expression of these two antigens (KEL*K and FY*A, respectively)^26^ (Fig. 1c,d).

In addition to the amplicons described above, additional amplicons targeting 99 common polymorphic loci were used as reference loci to determine expected molecular counts for each fetal antigen (see bioinformatic pipeline below and supplementary materials). Sequencing libraries were prepared by conducting one round of multiplexed PCR and one round of PCR-based indexing on 10 μL of purified cfDNA. Libraries were sequenced on an Illumina NextSeq 2000 sequencer with 1 × 101 sequencing; the fetal antigen loci and fetal fraction loci had an average read depth of 7200 reads/locus and 3900 reads/locus, respectively.

### Assay bioinformatic analysis

For each amplicon of interest, molecule counts are calculated using QCT technology as described previously (see Supplementary Materials for additional details)^22^. Briefly, QCT templates contain an embedded molecular identifier (EMI), which is added to the sample prior to PCR amplification. The post-amplification reads per molecule value is used compute the absolute detected number of pre-amplification molecules for the antigen of interest (ADM) and nine informative polymorphic loci are used to calculate absolute expected number of molecules (AEM). The number of detected molecules for an antigen of interest, ADM, divided by the number of expected molecules, AEM, is the calibrated fetal antigen fraction (CFAF).

For the RhD antigen five amplicons are used, CFAF is computed for each amplicon, and the second-highest measurement is used as the overall CFAF. This is a more conservative approach than using the median (the third-highest measurement) and has a negligible effect on the false positive rate while reducing the risk of false negatives that can be caused by amplification outliers. Such outliers can occur due to *RHD*/*RHCE* gene recombinations that lead to a single or multiple exon loss but still result in a RhD-positive phenotype, or when rare SNVs coincide with the primer regions.

The determination of antigen presence or absence is based on whether the CFAF is above or below predetermined thresholds, established based on the CFAF distributions of 8992 clinical plasma samples from pregnant individuals not used elsewhere in validation (Table S2). In an antigen-negative pregnant person, any cfDNA from that antigen would be of fetal origin and therefore antigen-positive and antigen-negative would have expected CFAFs of 100% and 0%, respectively. CFAF is corrected to normalize values around 100% for antigen-positive fetuses based on amplification efficiencies for individual antigen loci. The computed CFAF are categorized as fetal “antigen detected” and “not detected”. The assay employs a “no results” CFAF range (between the “detected” and “not detected” ranges) to prevent any false positives due to spurious issues such as sequencing noise, amplification errors, or contamination (Table S2).

The AEM thresholds for each antigen genotype were determined by initially modeling fetal molecules with a Poisson distribution l = AEM and n = 0 (for a minimum of 98% sensitivity even at the lowest molecular input samples) and then calibrated and verified through analytical validation (Table S2).

The fetal fraction is also reported as part of this assay. It is calculated from the same set of amplicons used to determine the AEM. The same locus selection process is used to select up to nine polymorphic loci in which a paternal allele was detected. The fetal fraction is calculated as two times the paternal allele fraction at the median locus. This approach has been described and validated previously^22–24^.

### Preparation of preclinical samples

Preclinical samples were created from twelve parent–child genomic DNA pairs with known antigen genotypes purchased from Coriell. At least one parent antigen-negative/child antigen-positive pair and one parent antigen-negative/child antigen-negative pair were created for each antigen to mimic the genotype combinations of clinical samples. To make the preclinical samples, DNA was sheared to an average size of 175 bp using a Covaris S220 focused-ultrasonicator to mimic cfDNA fragmentation. Sheared DNA was diluted to a concentration of 0.236 ng/μL or 0.471 ng/μL and the parent–child DNA was mixed to mimic fetal fractions ranging from 1.5% to 12%. Samples were replicated to create at least 93 samples for each antigen with an overweighting of lower fetal fractions to assure assay performance at low fetal fractions (Table S3).

### Selection and preparation of plasma samples from pregnant individuals with unknown fetal antigen genotypes and phenotypes

Three independent batches of clinical plasma samples from pregnant individuals with unknown fetal antigen genotypes or phenotypes were used in the development and initial validation of the NIPT assay. These samples were selected from deidentified saved plasma samples from pregnant individuals who had a negative genotype for RhD, C, c, E, K, or Fy^a^ antigens, which would be the genotype of an individual alloimmunized for that antigen. Samples were anonymized and included in the study regardless of the pregnant individual’s alloimmunity status, which was unknown. Samples were excluded if they did not meet initial quality control (QC) criteria, including: gestational age < 10 weeks or unknown, < 0.5 ng DNA, contamination detected, no sequencing data, or low read depth (< 200 average reads per locus or < 100 reads at any reported fetal antigen locus). The batch of 8992 clinical plasma samples was used to calibrate the assay (see assay overview and design, Table S2). A second batch of 15,500 plasma samples was used to validate the assay after initial validation on preclinical samples. For a third batch evaluating assay precision two plasma samples were analyzed independently from 1683 patients.

### Selection and preparation of plasma samples from pregnant individuals with known fetal antigen status

Clinical samples from pregnant individuals with known fetal antigen genotype and/or phenotype were obtained from two sources: (1) the LIFECODES biobank at Brigham and Women’s Hospital (Boston, MA) and (2) the UNITY fetal antigen patient registry study at BillionToOne.

LIFECODES, an IRB-approved biorepository, includes banked plasma samples from pregnant individuals (mean gestational age at entry, 11.2 weeks). Plasma samples were selected from LIFECODES that had been banked at – 80 °C for less than 2 years, were from serological RhD-negative pregnant individuals, and had known antigen serology in the resulting neonate.

Twenty-five RhD-negative pregnant person plasma samples were obtained from LIFECODES. Neonatal serology was blinded to the individuals performing the NIPT assay until the NIPT results for the samples were available. Libraries were sequenced on an Illumina MiSeq sequencer with 1 × 150 sequencing. The cfDNA fetal fraction was determined in the same manner described in the previous methods section.

Two of the 25 samples were excluded because they did not pass QC metrics: one due to very low fetal fraction and the other due to patient history of weak D phenotype (conflicting RhD-negative and RhD-positive clinical serology results). The remaining 23 samples were analyzed using the NIPT assay, including one sample with a fetal fraction of 1.1%, which would have been excluded in a clinical scenario because it was below the fetal fraction call threshold of 1.5%. After analysis, the neonatal serology was unblinded and results were assessed for the concordance of NIPT-determined fetal RhD status with neonatal RhD serology.

The UNITY fetal antigen patient registry study is an IRB-approved study for individuals who have undergone the fetal antigen NIPT assay as part of their clinical care. The study protocol involves the collection of cord blood samples or buccal swabs from the neonate from the corresponding pregnancy. The samples are de-identified and sent to Grifols laboratory for antigen genotyping on the FDA-approved ID CORE EX platform, which genotypes the sample for C, c, E, K, and Fy^a^ using the same SNVs as the NIPT assay^26^. Pregnant person and neonatal medical records, including antibody screens and titers and neonatal RhD serology, are collected to confirm pregnant person alloimmunization status and neonatal RhD phenotype. When neonatal RhD serology was unavailable, a separate assay was run at Grifols laboratory to sequence exons 1–10 of the *RHD* gene. Samples from 30 pregnant individuals alloimmunized to one or more antigens were included in the analysis. The fetal antigen NIPT results were compared to the Grifols-reported antigen genotypes and predictive phenotype to determine concordance.

### Statistical analysis

Sensitivity and specificity were computed for the analytical samples, biobank, and patient registry samples. Clopper-Pearson 95% confidence intervals were reported. For the clinical samples of unknown fetal antigen genotype, a model was fit to the CFAF values categorized as fetal antigen detected to estimate the sensitivity of the assay (the likelihood that a sample with fetal antigen detected by the NIPT assay represents a fetus positive for the given antigen genotype). The CFAF values for the antigen detected samples are not normally distributed because of the established CFAF thresholds for categorization of antigen detected. Therefore, a truncated normal distribution that accounts for the lower CFAF threshold resulted in the best fit for these data. Finally, we provide summary statistics, number of samples received, results reported and no results rate, of our clinical experience of the fetal antigen NIPT assay.

### Ethics declarations

Informed consent was obtained for all human research participants included in this publication. The study protocol was approved by the WCG IRB 20225380. All methods were carried out in accordance with relevant guidelines and regulations.

## Results

### Validation of the NIPT assay on preclinical samples

Analytical validation of the NIPT assay was performed on preclinical samples with established ground truth and a broad range of clinically relevant fetal fractions. Preclinical samples were made of genomic DNA from “parent” and “child” sources (see Preparation of preclinical samples, Table S3). NIPT for the detection of fetal RhD antigen genotype was first assessed with four parent–child DNA pairs mixed to mimic RhD-negative/RhD-positive and RhD-negative/RhD-negative pregnant person/fetus statuses at 12% fetal fraction. The fetal antigen NIPT assay demonstrated 100% accuracy on these samples for the identification of the fetal RhD antigen genotype (n = 96).

**Figure 2:**
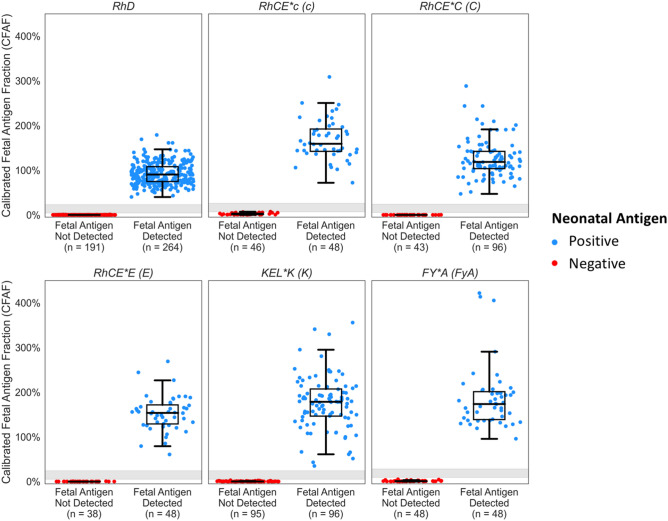
Calibrated fetal antigen fraction (CFAF) and identified fetal antigen status for preclinical samples. Samples were made from sheared genomic DNA samples from replicates of parent–child pairs with an antigen negative parent/ antigen positive fetus (blue) and antigen positive parent/antigen negative child (red) pairs for each antigen type (Table S1). Parent–child samples were mixed to mimic fetal fractions ranging from 1.5 to 12%. Row 1: RhD, RhCE*c and RhCE*C, Row 2: RhCE*E, KEL*K and FY*A. The grayed area on the graphs represents the antigen-specific CFAF intermediate range (Table S2). CFAF values in this range are reported as No Results and a new sample is requested for re-analysis. There were no discordant calls. There were 16 samples where no result was reported because there were too few molecules in the sample (absolute expected molecular (AEM) count below the antigen specific threshold, n = 11) or the CFAF value was within the intermediate range (n = 5). For all 1061 NIPT assays the identified fetal antigen status was concordant with the known fetal antigen status, resulting in an overall sensitivity of 100% (95% CI 99–100%) and specificity of 100% (95% CI 99–100%). The tabulations of these data are in Table 1.

To establish performance of the assay across a challenging range of fetal fractions, an additional 12 preclinical samples were made for the six antigens and replicated at fetal fractions from 1.5 to 12%, weighted toward lower fetal fractions to challenge the performance of the assay and demonstrate that the assay would make accurate calls at early gestational ages. The NIPT assay correctly determined fetal antigen status for all samples with an overall analytical sensitivity 100% (95% CI 99–100%) and specificity of 100% (95% CI 99–100%) (Fig. 2, Table 1). For 16 of the total 1,077 assays a call was not made; 11 due to too few fetal molecules (below the AEM threshold) and 5 where the CFAF fell within the intermediate zone. Fetal fraction was 3% for these 16 assays. Importantly, all 5 of the intermediate CFAF cases were fetal antigen negative, demonstrating the conservative design of the assay. The frequency of samples below the AEM threshold is higher than what would be experienced in clinical practice because the samples were weighted towards the minimum fetal fraction.

**Table 1:** Sensitivity, specificity of the NIPT assays on pre-clinical samples made from genomic DNA sheared and mixed to mimic cfDNA.

Antigen	Total^a^ (N)	NIPT fetal antigen detected (N)	NIPT fetal antigen not detected (N)	Sensitivity	95% CI	Specificity	95% CI
RhD	456	264	191	100%	(99–100%)	100%	(98–100%)
RhCE*C (C)	144	96	43	100%	(96–100%)	100%	(92–100%)
RHCE*c (c)	96	48	46	100%	(93–100%)	100%	(92–100%)
RHCE*E (E)	93	48	38	100%	(92–100%)	100%	(94–100%)
KEL*K (K)	192	96	95	100%	(96–100%)	100%	(96–100%)
FY*A (FyA)	96	48	48	100%	(93–100%)	100%	(93–100%)
Total fetal antigen	1077	600	461	100%	(99–100%)	100%	(99–100%)

^a^There were 16 samples where no results were issued due to low molecular count; absolute expected molecular (AEM) count below the antigen specific threshold (n=11) or calibrated fetal antigen fraction (CFAF) in the intermediate zone (n=5). Clopper–Pearson 95% confidence intervals (95% CI). Data are plotted in Fig. 2 and the pre-clinical samples are described in Table S3.

### Evaluation of the NIPT assay on clinical samples with unknown fetal antigen genotypes and phenotypes

**Figure 3:**
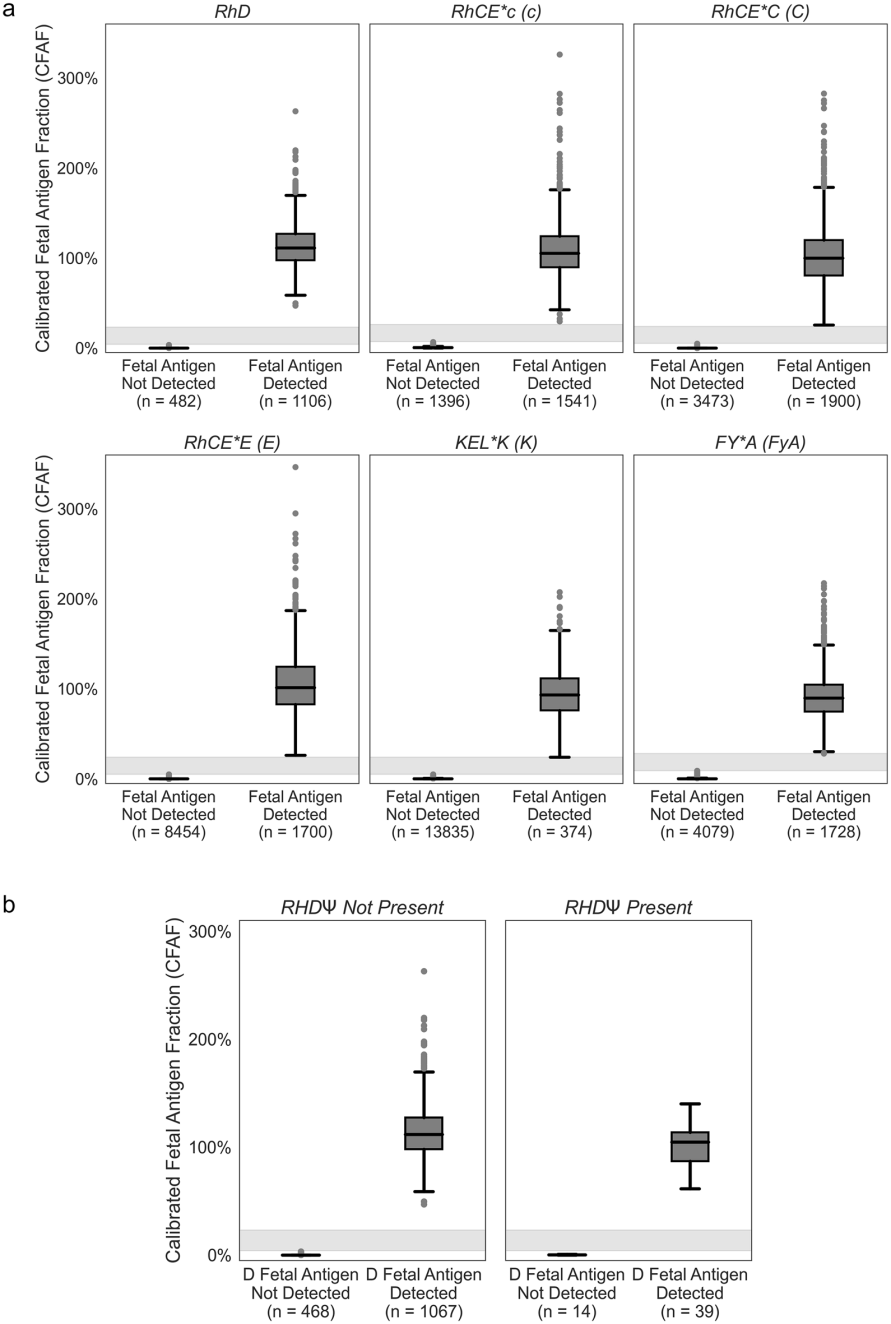
(**a**, **b**) Calibrated fetal antigen fraction (CFAF)s and identified fetal antigen status for clinical samples. Pregnant person antigen status was determined by NGS of cfDNA and fetal antigen status was reported for all samples where the pregnant person was identified to be negative for the antigen to mimic the clinical use of this assay. Samples are not unique across plots; the same sample was used for multiple assays if the pregnant person was antigen negative for multiple antigens. (**a**) Row 1: RhD, RhCE*C and RhCE*c, Row 2: RhCE*E, KEL*K and FY*A. There were 1191 (2.9%) NIPT analyses where fetal antigen was not determined due to low molecular count (absolute expected molecular (AEM) count below the antigen specific threshold, n = 941), CFAF in the intermediate zone (n = 180), or number of fetal antigen molecules detected above the expected range (n = 70). The sensitivity was modeled using a truncated normal distribution for the fetal antigen detected samples and the estimated sensitivity was greater than 99.6%. The tabulations of these data are in Table S5, the individual data points are plotted in Fig. S1a, data are plotted by fetal fraction in Fig. S1c, and the sensitivity of the antigen positive samples is modeled in Fig. S2. (**b**) The fetal RhD prediction for the 1615 clinical samples where the RhDΨ variant was not detected (panel 1) and the 53 clinical samples where RhDΨ variant was detected (panel 2). In qualitative RhD NIPT assays, samples with RhDΨ variant result in a no-call or inconclusive result. The individual data points are plotted in Fig. S1b.

Next, to demonstrate the ability of the assay to assess fetal antigen genotype on clinical plasma samples, NIPT was performed on plasma obtained from pregnant individuals (see Selection and preparation of clinical samples with unknown fetal antigen genotypes and phenotypes). Plasma samples from the clinical patients were analyzed for fetal antigen genotype for all antigens for which the pregnant person was genotype negative. This type of sample selection is consistent with the clinical application of the assay where alloimmunized pregnant persons are genotype-negative for the antigen(s) to which they are alloimmunized. This initial cohort included 15,500 saved plasma samples, of which 171 were excluded because they did not meet QC requirements. This resulted in 15,329 samples with an average gestational age of 13.9 weeks (range 10.0–39.0) and a fetal fraction of 8.8% (range 1.5–37.3%) (Table S4). There were 40,036 NIPT assays with an informative fetal antigen result on the 15,329 samples and the estimated sensitivity, modeled by analyzing the separation between positive and negative calls, was greater than 99.5% for all antigens (Table S5, Figs. 3a, S1a, S2; see “Methods”). As expected, the CFAF values were independent of fetal fraction (Fig. S1c).

There were 53 samples (3.3% of RhD-negative samples) for which the *RHDΨ* variant was present (as detected by the NIPT assay in the pregnant person and/or fetal cfDNA). The assay differentiates between fetal and maternal sources of non-deletion variants by examining whether the CFAF measurement is consistent with a maternal or fetal cell-free DNA levels. In 14 samples, the NIPT assay identified the fetus as RhD-negative, and in 39 the fetus was RhD-positive (Figs. 3b, Fig. S1b). These samples with the *RHDΨ* variant would have resulted in no results or incorrect fetal RhD phenotype prediction for other qualitative NIPT assays due to the inability to quantify the *RHDΨ* cfDNA and, therefore, predict fetal phenotype^20,27^.

There were 41,227 antigen calls attempted on the 15,329 samples and 1191 assays where fetal antigen was not determined for the following reasons: AEM below the antigen specific threshold (n = 941, 2.3%), intermediate CFAF value (n = 180, 0.44%), or CFAF value or number of fetal antigen molecules detected above the expected range for an antigen negative pregnant individual (n = 70, 0.16%). In a clinical scenario, the no result rate was lower because no-result assays were repeated on a backup plasma sample. For example, from this same 15,500 cohort, there were 711 cases where RhD NIPT was ordered clinically to assess fetal RhD status. All samples passed QC and all had an informative NIPT result (206 fetal antigen not detected, 505 fetal antigen detected) including 18 samples with *RHDΨ*; for an overall no results rate of 0%. Additionally, across 769 pregnant people alloimmunized for 889 antigens sent for clinical NIPT, an informative NIPT result was reported for 99.9% of the assays with a single no result issued for RhCE*C due to AEM below the C-antigen threshold (Table S6). For these 769 pregnant people, the average gestational age was 18.8 weeks (range 10–38 weeks) and fetal fraction was 11.6% (range 1.6–48%).

Importantly, in clinical practice, the assay was able to identify the fetal antigen status even when the incorrect test for alloimmunized antigen was ordered. In 11 cases, follow up confirmed the wrong antigen had been ordered by the clinician, and in 12 RhD cases weak D was suspected or confirmed.

Precision is another important metric that correlates with test accuracy. To evaluate analytical precision, the concordance of NIPT results for a unique set of 1683 retained plasma samples with two remaining samples (3366 samples) was assessed. As with the analysis on other clinical plasma samples, NIPT was completed for all antigens for which the pregnant individual was genotype-negative, resulting in a total of 3921 NIPT analyses. All but 5 NIPT results were concordant across the two NIPT assays, resulting in a precision of 99.9% (95% CI 99.7–100%) (Table S7).

### Clinical validation on biobank samples with known serology and/or fetal genotype results

Finally, NIPT was completed on 53 plasma samples where the fetal/neonatal antigen phenotype and or genotype was known to enable evaluation of the accuracy of the assay on clinical plasma samples. The 23 samples from the LIFECODES biobank and 30 samples from the UNITY fetal antigen patient registry study had fetal fractions ranging from 1.1 to 29.1% (Table S8). The NIPT-identified fetal antigen genotype was consistent with the known neonatal antigen genotype or phenotype for all samples.

**Figure 4:**
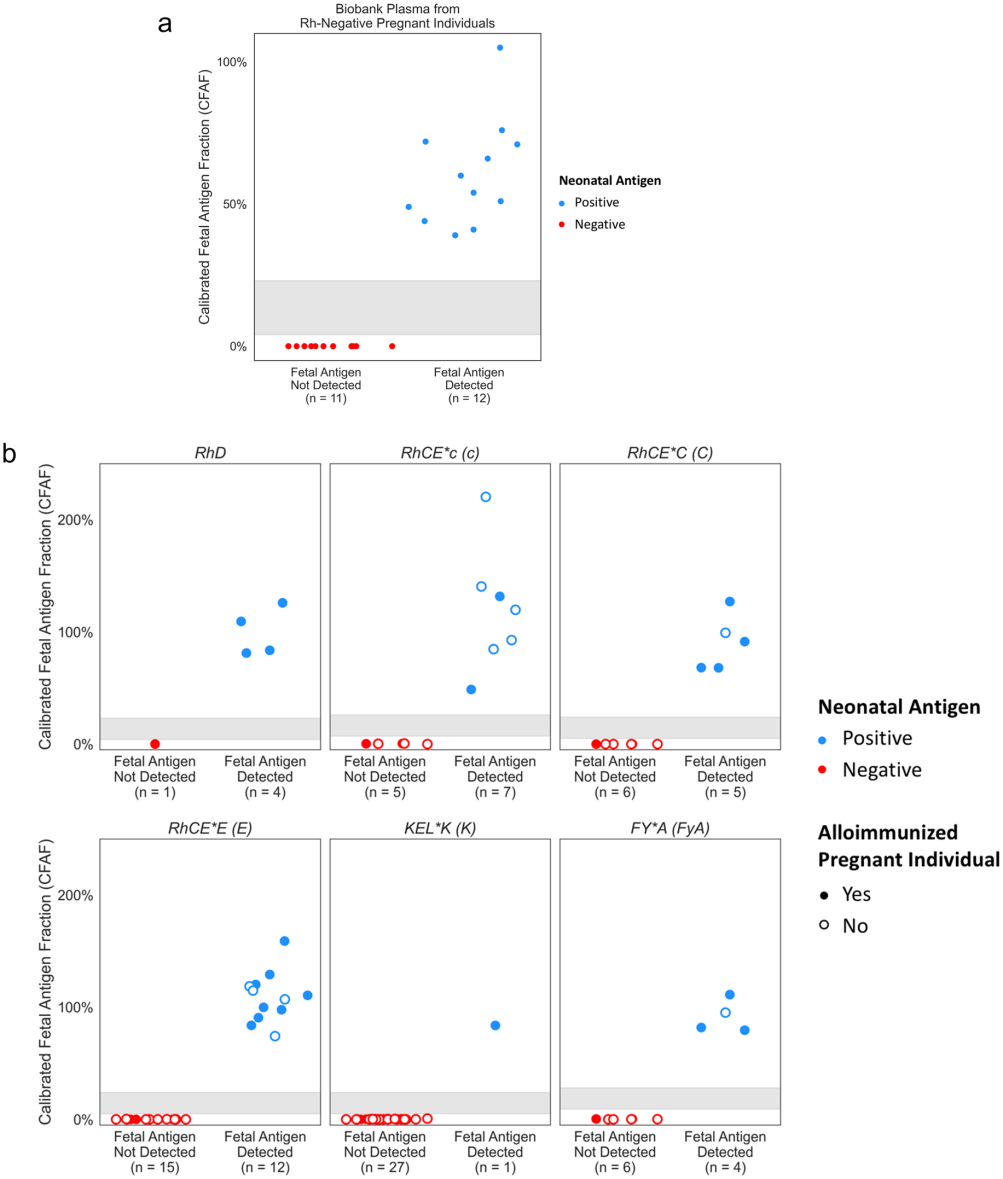
(**a**) CFAF is plotted for the 23 samples from the LIFECODES biobank with known fetal RhD serology. All NIPT results were concordant with neonatal serology. Neonate RhD-positive by postnatal serology is indicated in blue and neonate RhD-negative by postnatal serology is indicated in red. The sensitivity and specificity for the detection of fetal RhD based on these 23 biobank samples was 100% (95% CI 73.5–100.0%) and 100% (95% CI 71.5–100.0%). respectively. (**b**) CFAF plotted for the 30 samples from alloimmunized pregnant individuals for the 93 antigens the pregnant individual was alloimmunized to and/or had a negative genotype. All NIPT results were concordant with neonatal genotype. Neonate antigen positive by postnatal genotyping is indicated by blue and neonate antigen negative in postnatal genotyping is indicated in red. An unfilled dot indicates the pregnant person was alloimmunized for the antigen. For these 93 antigens, the NIPT for detection of fetal antigen had a sensitivity of 100% (95% CI 89.4–100.00%) and a specificity of 100% (95% CI 94.0–100.0%).

The concordance evaluation for the biobank samples included 12 fetuses correctly identified as RhD-positive and 11 fetuses correctly identified as RhD-negative, resulting in a clinical sensitivity of 100% (95% CI 73.5–100.0%) and a clinical specificity of 100% (95% CI 71.5–100.0%) for the detection of fetal RhD status (Fig. 4a).

The 30 samples from the UNITY fetal antigen patient registry included five individuals with anti-D, six with anti-C, four with anti-c, 11 with anti-E, six with anti-K, and five with anti-Fy^a^. There were seven individuals alloimmunized to more than one antigen. The Grifols antigen genotype assay, used to assess neonatal antigen genotype, can evaluate antigen genotypes for all antigens included on the NIPT panel, regardless of alloimmunization status. The NIPT assay can evaluate fetal antigen genotype for antigens where the pregnant person is genotype negative regardless of whether they are alloimmunized (in clinical practice, only ordered antigens are reported). Therefore, concordance was assessed for 93 antigen evaluations across the 30 samples, including the 37 antigens to which the pregnant person was alloimmunized. The NIPT-identified fetal antigen genotype and neonatal antigen genotype were concordant for all 93 evaluations resulting in a sensitivity of 100% (95% CI 89.4–100.0%) and specificity of 100% (95% CI 94.0–100.0%) (Fig. 4b).

## Discussion

This study presents the development and validation of an NIPT assay for fetal antigen detection appropriate for the diverse U.S. population with no partner sample required. NIPT for fetal antigen can decrease patient risk and anxiety, as well as healthcare burden, namely by reducing the administration of Rho(D) immune globulin, which is unnecessary for the up to 40% of RhD-negative pregnant individuals carrying an RhD-negative fetus. Moreover, for the approximately 1% of alloimmunized pregnant individuals, NIPT can prevent unnecessary fetal monitoring for the estimated 50–65% who are carrying an antigen-negative fetus^6^.

As ACOG has outlined, implementation of NIPT as standard of care for the U.S. pregnant population will require excellent detection to prevent sensitization in RhD-negative individuals and HDFN in alloimmunized pregnancies, high sensitivity for all ancestries to ensure feasibility and equity of care, and a cost-effective assay to limit healthcare burden^1,20^. These objectives have been achieved with this NIPT assay using QCT technology to detect fetal antigen status effectively and efficiently in the diverse U.S. population.

The use of cfDNA testing to guide administration of Rho(D) immune globin is standard of care in Denmark, the Netherlands, Belgium, England, Sweden, France, and Italy^4,6,7,10,18,27–30^. The reasons for this implementation include lower cost of care^31,32^, concerns about the potential risk for blood borne pathogens in Rho(D) immune globulin^33^, and lower adherence rates due to cultural beliefs when prophylaxis is recommended for all RhD-negative pregnancies^1^. The U.S. has not adopted a similar approach, likely due to the elevated rate of indeterminate results owing to non-*RHD* gene deletion variants, including *RHDΨ* and *Rh-CE-D* hybrid genes, which are more common in non-European individuals^16^. A previous study of RhD NIPT in a U.S. population had an overall indeterminant rate of 11%, with over half due to an *RHDΨ/RHD* gene variant^34^. In this study, the QCT technology-based quantitative approach with NGS enables the assay to detect fetal RhD status both for the common *RHD* gene deletion and other *RHD* variants, and therefore informative results were returned for 100% (no results rate 0%) of the clinically-ordered RhD NIPT assays with gestational ages as early as 10 weeks. The ability of this assay to perform accurately at early gestational ages is important given utility of RhD NIPT to guide RhD prophylaxis decisions when sensitizing events occur in the first trimester of pregnancy^35^. Additionally, the NIPT assay was able to determine fetal RhD antigen status for samples with the *RHDΨ* variant, which was present in 3.3% of all RhD-negative pregnant individuals. Conversely PCR-based assays cannot predict fetal RhD phenotype in the presence of a non-*RHD* gene deletion variant^8,13,19,34^. Moreover, the RhD NIPT assay had an analytical sensitivity of 100% (95% CI 99–100%) and specificity of 100% (95% CI 98–100%) and correctly identified the fetal antigen status for 23 biobank samples with known neonatal serology.

In current clinical practice, grey-zone phenotypes such as weak D and partial D, can result in inconsistent serology results, which can lead to patients not receiving appropriate Rho(D) immune globulin^36^. The current NIPT assay was designed to be robust with regards to these edge cases in order to minimize or eliminate false negative results. Specifically, the five-amplicon based assay is designed to return a fetal RhD-positive phenotype for rare *RHD* gene variants that are outside of the *RHDΨ* and *Rh-CE-D* hybrid genes. This design ensures these patients receive Rho(D) immune globulin, in line with recommended clinical practices for grey-zone phenotypes^36^.

Increasingly, outside the U.S., fetal antigen NIPT is already used to guide management for alloimmunized pregnancies and is standard of care in the UK^30^. European-based assays are unable to quantify fetal cfDNA and, therefore, require a later gestational age and sometimes repeat samples leading to unnecessary parental anxiety and costly pregnancy monitoring^19^ The assay described here was designed with an absolute expected fetal antigen molecule (AEM) threshold to maximize sensitivity and call rate at very low fetal fractions and, therefore, early gestational age. In the current study, the assay determined fetal antigen genotype in pregnancies as early as 10 weeks gestation. Furthermore, across the C, c, E, K and Fy^a^ antigens, the NIPT assay had an analytical sensitivity of 100% (95% CI 99–100%) and specificity of 100% (95% CI 99–100%) and correctly identified the fetal antigen status for 93 antigens among 30 alloimmunized pregnant individuals. In our clinical experience of the 889 assays with gestational age as early as 10 weeks and fetal fractions as low as 1.6%; an informative result was issued for 99.9% of assays. Furthermore, through quantitative assessment of the antigen present in cfDNA, the test was able to identify 11 cases where the incorrect antigen had been ordered, based on CFAF levels consistent with the pregnant person having the antigen genotype, allowing re-analysis for the correct antigen.

The intermediate no-call CFAF threshold used in alloimmunized cases, where a fetal antigen status is not reported and a new sample is run, maximizes the specificity of the assay. The frequency of an intermediate call is rare and did not occur in the 769 alloimmunized pregnant people. The strengths of the assay are further illustrated by two biobank samples that did not meet the inclusion criteria. Interrogation of the cfDNA NIPT sequencing data from the sample where the pregnant individual had conflicting RhD serology (suspected weak D) identified *RHD* gene variants (p.T201R and p.F223V) in the exon 4 amplified region, a known haplotype consistent with weak D type 4^16^. Additionally, for the other excluded sample which had a fetal fraction of only 0.15%, NIPT results were concordant with the neonatal serology, demonstrating that the QCT quantification of *RHD* gene molecules is robust at extremely low fetal fractions.

While the data demonstrate the accuracy and robustness of the assay, this study has not measured the impact of assay on clinical care and healthcare cost savings. However, given the assay’s performance, including lack of false negatives and low no-result rates comparable to or superior to currently recommended diagnostics, it is expected that the implementation into broad clinical care will improve standard of care, as has been demonstrated in European practice^31,37–39^.

Fetal genotyping of alleles for RhD, C, c, E, K, and Fy^a^ antigens is an important tool for managing sensitization in RhD-negative pregnancies and HDFN in alloimmunized pregnancies. In this study, a NIPT assay for RhD-negative individuals had a sensitivity and specificity of 100% (95% CI 99–100%), demonstrating robustness when utilized within the diverse U.S. population. When implemented into standard care this assay could streamline care by nearly eliminating unnecessary prophylactic treatment for approximately 40% of RhD-negative pregnancies. Furthermore, the NIPT assay for alloimmunized individuals correctly determined the fetal antigen status with a sensitivity and specificity of 100% (95% CI 99–100%). The accuracy of this approach indicates it may be more effective for identifying alloimmunized pregnancies at risk for HDFN than the current ACOG guidelines, which include testing the reproductive partner and invasive diagnostic testing to determine fetal antigen status^3^. The ACOG recommended approach is limited by up to 10% misattributed paternity, incomplete of partner testing, and poor adherence to invasive diagnostics when the partner is found to be heterozygous^40,41^. These factors do not impact NIPT as no partner sample is needed. Furthermore, management of alloimmunized pregnancies potentially at risk for HDFN is intensive and includes frequent middle cerebral artery (MCA) Doppler screening for detection of fetal anemia. MCA doppler screening has a risk for false positive findings, which can result in unnecessary invasive procedures^42^. The clinical implementation of NIPT can significantly improve the standard of care for both prevention of RhD alloimmunization and management of alloimmunization in pregnancy.

### Supplementary Information

Supplementary Information 1.

Supplementary Tables.

Supplementary Figures.

## Data Availability

The minimal dataset that would be necessary to interpret, replicate and build upon the findings reported in the article is available upon request from the corresponding author.
