# Supplementary material for: Validation of a non-invasive prenatal test for fetal RhD, C, c, E, K and Fya antigens
Source: Sci Rep. 2023 Aug 7;13:12786. doi: 10.1038/s41598-023-39283-3 (PMC10406947; doi:10.1038/s41598-023-39283-3)
Supplement: Supplementary file 1 — Supplementary Information 1. [file 41598_2023_39283_MOESM1_ESM.docx]

**Supplementary Methods**

Calibrated Fetal Antigen Fraction (CFAF) Computation

For the five amplicons for the RHD*D (RhD) antigen allele and the individual amplicons for RHCE*C (C), RHCE*C (c), RHCE*E(E), KEL*K(K), and FYA*A(FyA) antigen alleles, molecule counts are calculated using QCT technology as described previously [22]. First, QCT templates which contain an embedded molecular identifier (EMI), are added to the sample prior to PCR amplification. The conditions are such that each EMI can be assumed to be unique pre-amplification. The PCR reaction will amplify both endogenous sample DNA at the locus and the QCT templates at a given location. After NGS sequencing, the reads per EMI are calculated and have been shown to correlate to the reads per pre-amplification endogenous sample molecule [22]. The reads per molecule value is used to convert the number of NGS sequencing reads to the absolute detected number of pre-amplification molecules for the antigen of interest (ADM) present in the original sample.

In addition to molecule counts for the antigen of interest, molecule counts at reference loci containing paternal alleles are also calculated. Ninety-nine loci throughout the genome with two common SNPs (each at approximately 50% allele frequency) were chosen to maximize the likelihood of detecting a paternal allele. Paternal alleles are expected at approximately 25 of these loci. The number of pre-amplification paternal molecules is calculated using QCTs at each of these locations. To reduce variability and outlier effects, the loci with a detected paternal allele are sorted by read depth, and the median of the paternal allele molecular counts from the highest read dept loci (up to 9 loci in total) is used as the absolute expected number of molecules (AEM). The AEM is the number of molecules that would be expected to be found in the sample if the fetus had a paternal allele at a location of interest, and is used as a comparator of the ADM.

The number of detected molecules for an antigen of interest, ADM, divided by the number of expected molecules, AEM, is used to make a call for whether the antigen is present in the sample.

This measurement is referred to as the calibrated fetal antigen fraction (CFAF).

$$calibrated fetal antigen fraction \left( CFAF \right)=\left( \frac{ADM}{AEM} \right)$$
