# Supplementary material for: Validation of a non-invasive prenatal test for fetal RhD, C, c, E, K and Fya antigens
Source: Sci Rep. 2023 Aug 7;13:12786. doi: 10.1038/s41598-023-39283-3 (PMC10406947; doi:10.1038/s41598-023-39283-3)
Supplement: Supplementary file 2 — Supplementary Tables. [file 41598_2023_39283_MOESM2_ESM.docx]

| **Supplementary Table 1.** Primer sequences for the 5 amplicons in the *RHD* assay and the single amplicons to detect the SNVs associated with the other antigens. | | | |
| --- | --- | --- | --- |
| **Antigen** | **Amplicon Name** | **Forward Sequence** | **Reverse Sequence** |
| RhD | RHD Exon 4-1 | ctttctccaaggactatcagggctt | aggctgcgaacacgtagatgtgcatcat |
| RhD | RHD Exon 4-2 | tggtgcctgccaaagcctcta | cagcatggcagacaaactgggta |
| RhD | RHD Exon 5 | tgtggatgttctggccaagt | gctacagcatagtaggtgttgaaca |
| RhD | RHD Exon 7 | ccacagctccatcatgggct | aagcccagtgacccacatgcca |
| RhD | RHD Exon 10 | tcctctcactgttgcctgcatttgt | agaaaggattcaactccattttctctgact |
| RhCE*C (C) | RHCE-C intron 2 | ctcattgctatagcttaaggactca | gcagaggctgcaatgagctat |
| RhCE*c (c) | RHCE Exon 2 | gtgtgcagtgggcaatcct | tgaacagtgtgatgaccacct |
| RhCE*E (E) | RHCE Exon 5 | tgtggatgttctggccaagt | gctagagcatagtaggtgttgaaca |
| KEL*K (K) | Kell | ctgaagaaagggaaatggcca | gcgcatctctggtaaatgga |
| FY*A (FyA) | Duffy Antigen | tcttcctatggtgtgaatgattcct | cagctgcttccaggttgg |

| **Supplementary Table 2.** Calibrated fetal antigen fraction (CFAF) detected and not detected ranges were determined through analysis of 8,992 clinical samples. CFAFs that fall between detected and not detected ranges are reported out as a no call and in a clinical scenario a new sample is requested. Absolute expected fetal molecule (AEM) threshold was determined by Poisson distribution where at the lowest AEM the sensitivity does not fall below 98%. | | | | |
| --- | --- | --- | --- | --- |
| **Antigen** | **CFAF Antigen Not Detected Range** | **CFAF Antigen Detected Range** | **Fetal Fraction Automated Report Range**** | **AEM Threshold** |
| RhD | <4.0% | >23% | >1.5% | >2 |
| RhCE*C (C) | <5.0% | 24-300% | >1.5% | >6 |
| RhCE*c (c) | <7.0% | 26-300% | >1.5% | >4 |
| RhCE*E (E) | <5.0% | 24-300% | >1.5% | >6 |
| KEL*K (K) | <5.0% | 24-240% | >1.5% | >4 |
| FY*A (FyA) | <9.0% | 28-300% | >1.5% | >4 |
| Abbreviations: calibrated fetal antigen fraction (CFAF), absolute expected fetal molecule (AEM) | | | | |

| **Supplementary Table 3.** Coriell identities of the contrived parent-child samples used for clinical validation. Samples were mixed to at various concentrations to mimic fetal fraction cfDNA samples ranting from 1.5-12%. | | | | | |
| --- | --- | --- | --- | --- | --- |
| **Sample Type** | **"Pregnant Person" Status** | **"Pregnant Person" Coriell ID** | **"Fetus" Status** | **"Fetus" Coriell ID** | **Total Number of Samples** |
| RhD negative | RhD -/- | HG01620 | RhD -/- | HG01621 | 48 |
| RhD positive | RhD -/- | NA19131 | RhD +/- | NA19132 | 48 |
| RhD negative | RhD -/- | NA06989 | RhD -/- | NA12490 | 72 |
| RhD negative | RhD -/- | NA12273 | RhD -/- | NA10837 | 72 |
| RhD positive | RhD -/- | NA12156 | RhD +/- | NA10831 | 108 |
| RhD positive | RhD -/- | NA12004 | RhD +/- | NA10838 | 108 |
| RhCE*c negative | RhCE*c -/- | HG00590 | RhCE*c -/- | HG00591 | 48 |
| RhCE*c positive | RhCE*c -/- | HG00566 | RhCE*c +/- | Hg00567 | 48 |
| RhCE*C negative | RhCE*C -/- | NA19131 | RhCE*C -/- | NA19132 | 48 |
| RhCE*C positive | RhCE*C -/- | HG00565 | RhCE*C +/- | HG00567 | 48 |
| RhCE*E negative | RhCE*E -/- | NA19656 | RhCE*E -/- | NA19654 | 48 |
| RhCE*E positive | RhCE*E -/- | HG00566 | RhCE*E +/- | HG00567 | 48 |
| KEL*K negative | K -/- | HG00565 | K -/- | HG00567 | 96 |
| KEL*K positive | K -/- | NA19656 | K +/- | NA19654 | 96 |
| FY*A negative | Fy^a^ -/- | HG01173 | Fy^a^ -/- | HG01175 | 48 |
| FY*A positive | Fy^a^ -/- | HG01522 | Fy^a^ +/- | HG01523 | 48 |

| **Supplementary Table 4.** Characteristics of the 15,329 clinical plasma samples where the pregnant person was negative for at least one antigen of interest. | | |
| --- | --- | --- |
|  | **Mean** | **Range** |
| Pregnant Individual's Age | 28.9 | 12.0-54.0 |
| Gestational Age (weeks) | 13.9 | 10.0-39.0 |
| Fetal Fraction | 8.8% | 1.5%-37.3% |
| Race and Ethnicity^ |  |  |
| White, Non-Hispanic | 4777 | 45.3% |
| Black, Non-Hispanic | 2035 | 19.3% |
| Hispanic | 2943 | 27.9% |
| Asian | 379 | 3.6% |
| Other | 902 | 8.5% |
| Unknown | 4293 |  |
| Total analyzed for each antigen# |  |  |
| RhD | 1615 | 10.5% |
| RhCE*C (C) | 5706 | 37.2% |
| RhCE*c (c) | 2977 | 19.4% |
| RhCE*E (E) | 10549 | 68.9% |
| KEL*K (K) | 14463 | 94.4% |
| FY*A (FyA) | 5917 | 38.6% |
| ^Out of the 10,552 where race or ethnicity were known, N and % | | |
| #Only samples where the pregnant person was antigen negative by next generation sequencing were analyzed to mimic clinical use of the assay, N and % | | |

| **Supplementary Table 5.** 15,329 retained clinical samples. Proportion of pregnant people negative for each antigen based on NGS of cfDNA. Samples are not unique; the same sample was used for multiple assays if the pregnant person was antigen negative for multiple antigens. Samples for each antigen did not pass the initial quality control were not analyzed (n=371). For the samples analyzed by fetal antigen NIPT, proportion of fetal antigen detected, fetal antigen not detected and modeled mean, standard deviation, and sensitivity from truncated normal distribution fit to the NIPT CFAF values for the fetal antigen detected samples. | | | | | | |
| --- | --- | --- | --- | --- | --- | --- |
| **Antigen** | **N^** | **% NIPT Fetal Antigen Not Detected** | **% NIPT Fetal Antigen Detected** | **Modeled Mean CFAF (expected mean 100%)** | **Modeled CFAF Standard Deviation** | **Modeled Sensitivity** |
| RhD | 1615 | 29.8% | 68.5% | 112.9% | 24.6% | 100% |
| RhCE*C (C) | 5706 | 60.9% | 33.4% | 107.8% | 29.5% | 100% |
| RhCE*c (c) | 2977 | 46.9% | 52.0% | 90.0% | 25.7% | 99.9% |
| RhCE*E (E) | 10549 | 80.1% | 16.1% | 93.1% | 30.6% | 99.8% |
| KEL*K (K) | 14463 | 95.7% | 2.6% | 103.4% | 36.7% | 99.6% |
| FY*A (FyA) | 5917 | 68.9% | 29.5% | 99.5% | 32.4% | 99.8% |
| ^There were 1191 (2.9%) NIPT analyses where no results were issued due to low molecular count; absolute expected molecular (AEM) count below the antigen specific threshold (n=941), CFAF in the intermediate zone (n=180), or number of fetal antigen molecules detected was above the expected range (n=70). Data are plotted in Figure 3ab. | | | | | | |
| Abbreviations: non-invasive prenatal testing (NIPT), cell free DNA (cfDNA), next generation sequencing (NGS), calibrated fetal antigen fraction (CFAF), absolute expected molecular count (AEM) | | | | | | |

| **Supplementary Table 6.** Clinical evaluation of fetal antigen NIPT from 769 alloimmunized pregnant individuals. | | | | | |
| --- | --- | --- | --- | --- | --- |
| **Antigen** | **N^** | **Quality Control Fail** | **No Results** | **NIPT Fetal Antigen Not Detected** | **NIPT Fetal Antigen Detected** |
| RhD | 200 | 0 | 0 | 47 | 153 |
| RhCE*C (C) | 95 | 0 | 1 | 46 | 48 |
| RhCE*c (c) | 78 | 0 | 0 | 25 | 53 |
| RhCE*E (E) | 225 | 0 | 0 | 135 | 90 |
| KEL*K (K) | 260 | 0 | 0 | 224 | 36 |
| FY*A (FyA) | 31 | 0 | 0 | 14 | 17 |
| ^There were 12 samples sent for RhD, 4 samples sent for RhCE*C, 3 samples sent for RhCE*c, and 4 samples sent for RhCE*E where the AEM indicated the pregnant individual was positive for the antigen and therefore NIPT was not indicated. | | | | | |
| Abbreviations: non-invasive prenatal testing (NIPT) | | | | | |

| **Supplementary Table 7.** 1683 retained clinical cases with two samples. Samples are not mutually exclusive. Concordance of independent fetal antigen NIPT analysis run on each sample duo stratified by samples that were concordant for fetal antigen genotype positive and fetal antigen genotype negative. The direction of the one discordant sample is unknown as the true fetal antigen genotype is unknown for these samples. | | | |
| --- | --- | --- | --- |
| **Antigen** | **Concordant N** | **Discordant N** | **% Agreement** |
| RhD | 168 | 0 | 100.0% |
| RhCE*C (C) | 528 | 1 | 99.8% |
| RhCE*c (c) | 276 | 0 | 100.0% |
| RhCE*E (E) | 950 | 3 | 99.7% |
| KEL*K (K) | 1370 | 0 | 100.0% |
| FY*A (FyA) | 629 | 1 | 99.8% |

| **Supplementary Table 8.** Characteristics of the 53 (23 LIFECODES biobank; 30 BTO research) samples with known neonatal antigen serology (RhD) (biobank) or genotype (research) | | | | |
| --- | --- | --- | --- | --- |
|  | **Mean** | **Range** | |  |
| **Pregnant Individual's Age** | 31 | (21-44) | |  |
| **Gestational Age** | 18.3 | (8.9-35.9) | |  |
| **Fetal Fraction** | 10.6% | (1.1%-29.1%) | |  |
| **Race and Ethnicity** | **N** | **%** | |  |
| Black, Non-Hispanic | 5 | 9.4% | |  |
| Mixed, Non-Hispanic | 2 | 3.8% | |  |
| Other, Hispanic | 7 | 13.2% | |  |
| South Asian, Non-Hispanic | 3 | 5.7% | |  |
| White, Non-Hispanic | 35 | 66.0% | |  |
| Not documented | 1 | 1.9% | |  |
| **Pregnant Individual's Alloimmunization Status** | | |  | |
| Not Alloimmunized; RhD-negative | 23 |  | |  |
| Anti-D | 5 | 16.7% | |  |
| Anti-C | 6 | 20.0% | |  |
| Anti-c | 4 | 13.3% | |  |
| Anti-E | 11 | 36.7% | |  |
| Anti-K | 6 | 20.0% | |  |
| Anti-FyA | 5 | 16.7% | |  |
