# Supplementary material for: Validation of a non-invasive prenatal test for fetal RhD, C, c, E, K and Fya antigens
Source: Sci Rep. 2023 Aug 7;13:12786. doi: 10.1038/s41598-023-39283-3 (PMC10406947; doi:10.1038/s41598-023-39283-3)
Supplement: Supplementary file 3 — Supplementary Figures. [file 41598_2023_39283_MOESM3_ESM.docx]

**Supplementary Figure 1a.b.c** Calibrated fetal antigen fraction (CFAF)s and predicted fetal antigen status for clinical samples with individual data points. The data in panels a. and b. are plotted in the condensed form in Figure 3ab. The data are tabulated in Table S5. **a.** Row 1: RhD, RHCE*C and RHCE*c, Row 2: RHCE*E, KEL*K and FY*A.


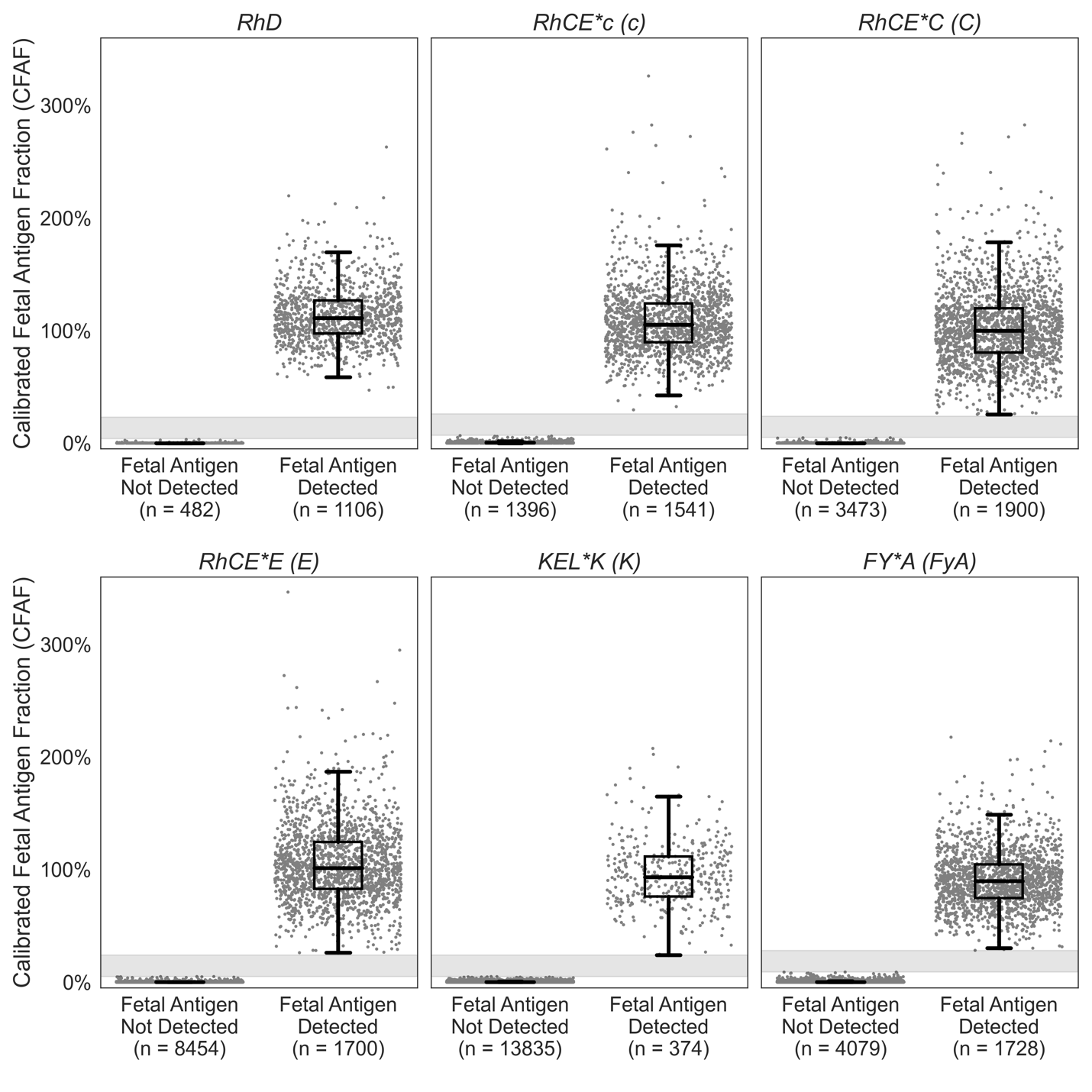


**b.** The fetal RhD prediction for the 1561 without clinical samples where the RhDΨ variant was not detected (panel 1) and the 56 clinical samples where RhDΨ variant was detected (panel 2). In qualitative RhD NIPT assays, samples with RhDΨ variant result in a no-call or inconclusive result.


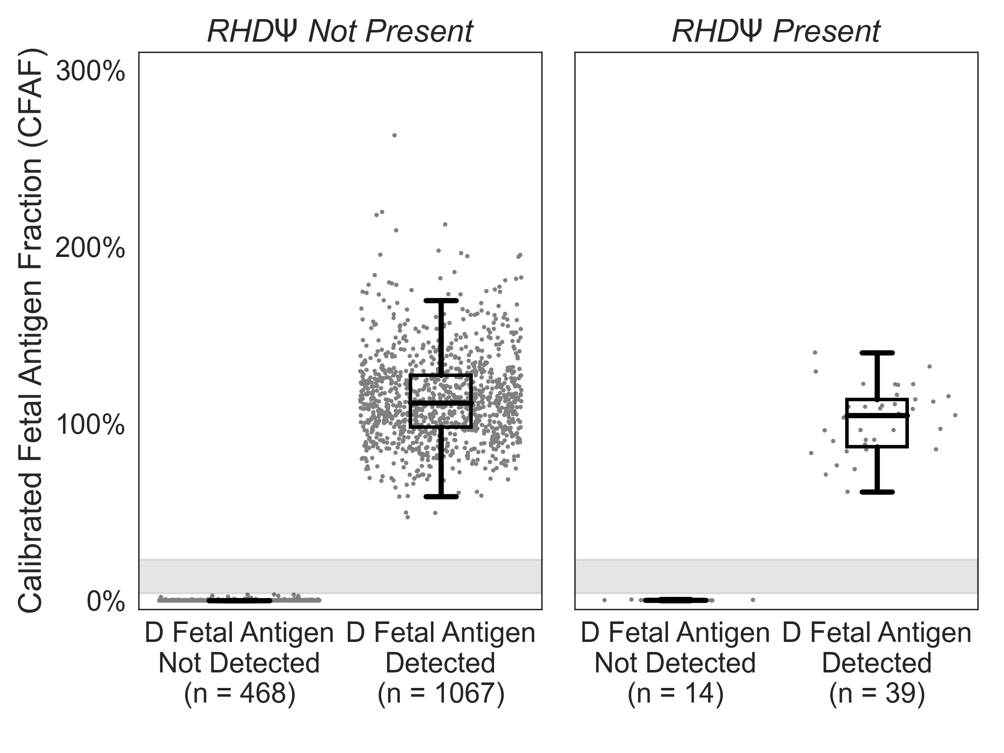


**c.** The data in **a.** plotted by fetal fraction.


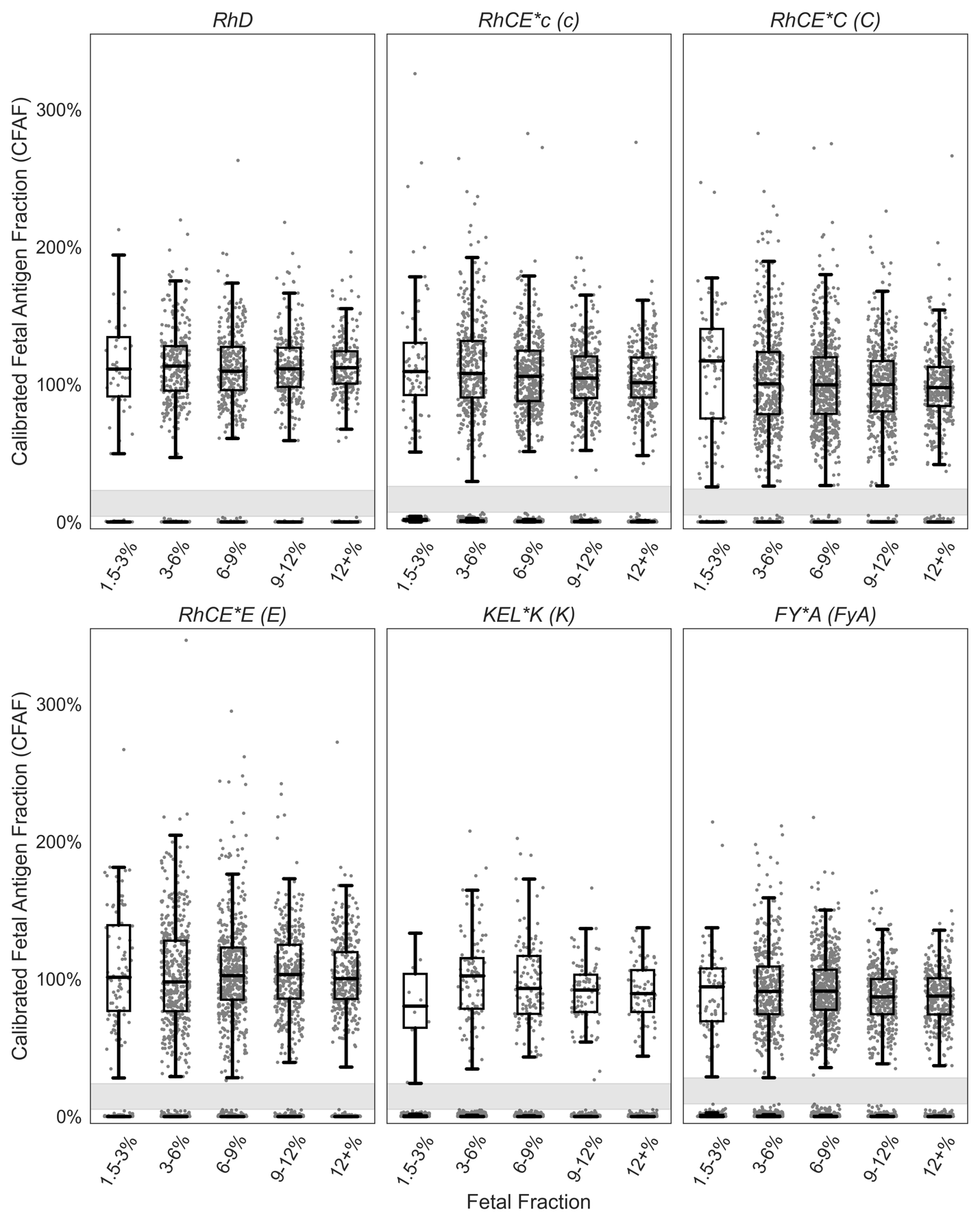


**Supplementary Figure 2.** Truncated normal distribution fit to the NIPT CFAF values categorized as antigen detected from 15,393 plasma samples from pregnant individuals of unknown fetal antigen genotype and unknown pregnant person alloimmunization status who were negative (by genotype) for at least one of the RBC antigens. The modeled sensitivity is in Table S5.
